# Iatrogenic Liver Perforation During Liposuction: A Case Report and In-depth Review of Clinical Presentation, Management, and Lessons Learned

**DOI:** 10.1093/asjof/ojad114

**Published:** 2023-12-21

**Authors:** Nasrin Jafarian, Rand Y Omari, Aldana E Shahbik, Omar Braizat, Mohammed Muneer

## Abstract

Liposuction is generally recognized as a safe medical procedure. However, it is important to acknowledge the potential for complications during and after the operation. Although rare, the occurrence of iatrogenic liver perforation following liposuction is viewed as a serious complication, necessitating immediate and attentive medical care. We report a case of a 42-year-old female who underwent liposuction and presented with abdominal pain 3 days later. Elevated liver enzymes and imaging revealed an active bile leak from the right liver lobe. Exploratory laparotomy confirmed a penetrating injury, leading to multiple washout surgeries. After a 3-month hospital stay, including intensive care, the patient fully recovered upon discharge following abdominal wound closure. Despite considering liposuction procedures safe due to the associated overall low risk rates, it can lead to life-threatening complications such as hollow viscus or solid organ injury. The treatment for such complications can either be surgical or nonsurgical, depending on the patient's presentation and diagnosis. To promptly identify and address any complication postsurgery, close monitoring of patients postoperatively is necessary.

**Level of Evidence: 5:**

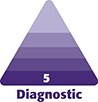

Liposuction is a widely spreading cosmetic body contouring procedure that targets subcutaneous fat tissues through suctioning cannulas to reduce the subcutaneous fat layer and provide enhanced cosmetic appearance.^1^ Common liposuction-related complications include contour irregularities, fluid accumulation, and pulmonary embolism.^2^ Rarely, complications of hollow viscus or solid organ injuries such as liver perforation and spleen injuries can arise and lead to life-threatening predicaments.^3^

According to several studies, the incidence of postliposuction iatrogenic liver perforation is considered very low, ranging from 0.02% to 0.09%.^3^ Although it is considerably rare, iatrogenic liver perforation is a critical life-threatening complication often requiring immediate medical attention and intervention. This case report provides an overview of this complication focusing on the incidence, risk factors, and management of postliposuction iatrogenic liver perforation.

The study was approved by the Institutional Review Board at Hamad Medical Corporation. Informed consent was obtained from the patient for publication of this case and accompanying images.

## CASE PRESENTATION

A 42-year-old female presented to our emergency department with abdominal pain and generalized body weakness 3 days after undergoing revision suction-assisted abdomino-dermolipectomy (SADL) with buttock lipo-filling and back dermolipectomy in a private hospital. The patient was healthy with no known comorbidities and was not on any regular medications. Her surgical history included a sleeve gastrectomy in 2015; laparoscopic cholecystectomy in 2016; and abdominoplasty, brachioplasty, and thigh lift in 2016.

Upon presentation, her vital signs were stable. Abdominal examination revealed severe diffused tenderness over the abdomen, mostly prominent in the left and right flanks, with associated rigidity. Laboratory results were remarkable for slightly elevated liver enzymes, elevated alkaline phosphatase, and elevated C-reactive protein of 200. The hemoglobin level was noted to be 10.2, and white blood cell count was within the normal range.

**Figure 1.**
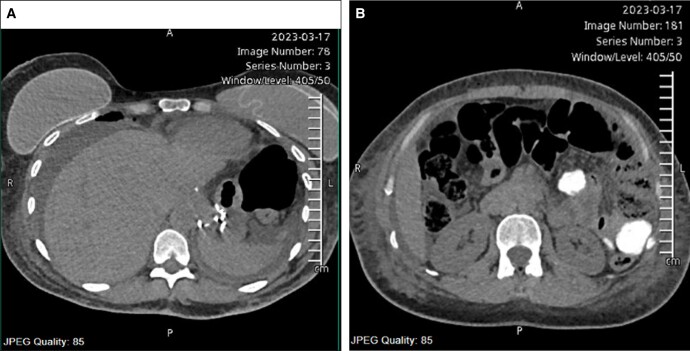
(A, B) Computed tomography scan showing active bile leak lateral to the right liver lobe.

MRI and computed tomography (CT) scan studies were performed, which showed an active bile leak lateral to the right liver lobe (hepatic segment 6) extending to the sub-hepatic region. An endoscopic retrograde cholangiopancreatography scan was also performed, revealing delayed passage of contrast into the distal common bile duct and mild intrahepatic biliary tree dilatation. However, there was no definite active contrast extravasation to suggest active bleeding (Figure 1).

The patient was admitted to the hospital and underwent exploratory laparotomy with peritoneal lavage. Intraoperatively, a penetrating injury was noted on the lateral surface of the right lobe of the liver, with an active bile leak detected from the site of the penetrated area. About 3 L of intraabdominal clear bile was drained, mainly from the right paracolic gutter, left paracolic gutter, and pelvis. The small bowel, large bowel, and other abdominal organs were intact with no visible or suspected perforation injuries. The patient underwent multiple washout surgeries and drain insertions, until no further bile leakage was noted. The patient required admission to the intensive care unit (ICU) for hemodynamic monitoring and organ support following initial exploratory laparotomy and remained in the ICU for 23 days due to hemodynamic instability and sepsis. The hospital course was also complicated by multiple episodes of metabolic derangement.

Following multiple washout surgeries, the abdominal wound was kept open, and a trial for abdominal wall closure showed that the fascia had retracted and was insufficient to achieve direct closure. Abdominal wall reconstruction was done through posterior component separation with transversus abdominis release and sublay mesh placement to allow for abdominal wall closure, and negative pressure wound therapy application was done to reduce the surface area of the open wound in the lower abdomen. Finally, split-thickness graft was done to close the remaining raw area of the lower abdomen measuring 35 × 12 cm. The patient remained in the hospital for a total of 3 months, including her stay in the ICU. Upon discharge, the patient was in good condition and had a complete recovery.

## DISCUSSION

Complications related to liposuction procedures are rising.^4^ These complications typically include thromboembolism, sepsis, and lidocaine toxicity.^5^ Fat embolism is also a relatively common complication after a liposuction procedure, with between 20% and 40% associated mortality rates.^6^ Complications, including abdominal organ perforations, are life-threatening and can arise, although rare, with an estimated occurrence rate of 0.014%.^7^

Iatrogenic liver perforation postliposuction is a serious complication that poses an immediate life-threatening risk.^5^ Intraabdominal perforations can be associated with the presence of diastasis of the rectus abdominis muscle, abdominal distension, and the presence of an underlying hernia.^8^ Several factors may also increase the risk of iatrogenic liver perforation during liposuction, such as the wrong trajectory when performing back-and-forth strokes during the procedure.^5^ The trajectory should be tangential, and the cannula should not be oriented deeply or in the direction of the viscera.^8^

Adhering to high operation standards is essential to mitigate the risks and conduct the procedure safely. A full preoperative assessment should be done to minimize the risk associated with iatrogenic perforation including medical and surgical history and complete physical examination.^8^ Proper patient selection is advisable prior to any surgical procedure. The physical examination should be complemented with imaging studies, such as ultrasound, in cases of previous abdominal surgeries or if herniation is suspected.^8^ It is crucial to optimize safe surgical techniques and maintain good orientation of the cannula during the procedure.

A high index of suspicion is needed to detect any solid or hollow organ injury postabdominal wall liposuction, as they can often be missed. Close monitoring of any patient undergoing liposuction is essential in the first two postoperative hours.^9^ Early signs that can facilitate detecting iatrogenic perforations include severe abdominal pain, fever, and low blood pressure.^10^ Signs of organ injury in the first 72 h include: abdominal pain and distension, nausea, vomiting, tachycardia, and hypotension.^11^ Patients undergoing the procedure should be educated to seek immediate medical attention if similar symptoms occur. If a patient presents with peritonitis or sepsis, blood workup such as complete blood count, metabolic panel, and inflammatory markers is recommended. In cases of suspected perforation, CT scans are considered adequate to establish an initial diagnosis.^12^ Contrast-enhanced ultrasound has been proven to be the preferred tool for monitoring penetrating liver injury because it permits a dynamic visualization of the ultrasound contrast agent wash over time to provide different vascular phases when examining the liver.^13^

Early diagnosis and prompt management are crucial to reduce morbidity and mortality associated with iatrogenic liver perforation. Depending on the severity of the liver injury, multiple lines of management may be utilized.^14^ Pohlan et al state that it is important to grade the severity of liver injury based on the criteria provided by the American Association of Surgery for Trauma, adding that an accurate diagnosis can facilitate efficient targeted surgical or nonsurgical treatment.^5^ Minor injuries may be managed conservatively with observation and supportive care, while more severe injuries may require angioembolization or other surgical interventions. It is recommended to involve a hepatobiliary surgeon in managing such injuries early on or have a multidisciplinary team to tackle such complications.

## CONCLUSIONS

This case report highlights a case of iatrogenic liver perforation postliposuction. Iatrogenic liver perforation is considered a rare but potentially life-threatening complication associated with liposuction procedures. Surgeons should be attentive to this complication, as early signs of iatrogenic perforation are easily missed and the consequences can lead to patient's mortality. A high index of suspicion, early diagnosis, and prompt management are crucial to achieve the best potential outcome for patients presenting with such complication. Surgeons should be aware of this potential complication and adequately take appropriate precautions to minimize the risk of iatrogenic liver injuries during liposuction procedures.
